# Waterborne Intumescent Fire-Retardant Polymer Composite Coatings: A Review

**DOI:** 10.3390/polym16162353

**Published:** 2024-08-20

**Authors:** Yang Li, Cheng-Fei Cao, Zuan-Yu Chen, Shuai-Chi Liu, Joonho Bae, Long-Cheng Tang

**Affiliations:** 1 Key Laboratory of Organosilicon Chemistry and Material Technology of Ministry of Education, College of Material, Chemistry and Chemical Engineering, Hangzhou Normal University, Hangzhou 311121, China; ly@hznu.edu.cn (Y.L.); chengfeicao168@gmail.com (C.-F.C.); chenzuanyu@stu.hznu.edu.cn (Z.-Y.C.); 2023112009039@stu.hznu.edu.cn (S.-C.L.); 2 Department of Physics, Gachon University, Seongnam-si 13120, Gyeonggi-do, Republic of Korea

**Keywords:** fire retardancy, intumescence, waterborne coating, polymer, environmental protection, safety

## Abstract

Intumescent fire-retardant coatings, which feature thinner layers and good decorative effects while significantly reducing heat transfer and air dispersion capabilities, are highly attractive for fire safety applications due to their effective prevention of material combustion and protection of materials. Particularly, the worldwide demand for improved environmental protection requirements has given rise to the production of waterborne intumescent fire-retardant polymer composite coatings, which are comparable to or provide more advantages than solvent-based intumescent fire-retardant polymer composite coatings in terms of low cost, reduced odor, and minimal environmental and health hazards. However, there is still a lack of a comprehensive and in-depth overview of waterborne intumescent fire-retardant polymer composite coatings. This review aims to systematically and comprehensively discuss the composition, the flame retardant and heat insulation mechanisms, and the practical applications of waterborne intumescent fire-retardant polymer composite coatings. Finally, some key challenges associated with waterborne intumescent fire-retardant polymer composite coatings are highlighted, following which future perspectives and opportunities are proposed.

## 1. Introduction

Fire-retardant coatings have developed as a mainstream fire safety technology both academically and commercially since the 20th century [1,2,3,4,5]. The evolution of fire-retardant coatings has advanced from early inorganic materials to intumescent formulations and modern waterborne, halogen-free systems [6]. Recently, nanotechnology and multifunctional coatings have significantly enhanced their performance, broadening applications in fire protection, corrosion resistance, and UV shielding [7]. Due to their highly effective fire protection and straightforward processing, they have been widely applied to the protection of materials such as metals, polymers, textiles, and wood [7,8,9,10,11]. Currently, fire-retardant coatings are categorized into intumescent and non-intumescent coatings based on their flame-retardant mechanisms [12]. Non-intumescent fire-retardant coatings, typically ceramic-based, achieve fire retardancy and thermal insulation by minimizing the heat transfer through the coating [13,14]. In contrast, intumescent fire-retardant coatings form an expanded char layer upon heating, providing thermal insulation and reducing heat and mass transfer between the condensed and gas phases [15,16,17]. In addition to excellent fire protection, intumescent coatings offer benefits such as weather durability, anti-corrosion capabilities, and decorative properties [18,19,20,21].

Intumescent coatings are classified into solvent-based and waterborne types based on the characteristics of the dispersion medium [22,23]. Traditional solvent-based intumescent coatings, despite their excellent fire protection performance, release large amounts of volatile organic compounds (VOCs) during production and application, causing environmental pollution and posing health risks [24,25]. Additionally, the high cost of organic solvents increases production costs, limiting their practical applications [26]. For environmental reasons, many solvent-based intumescent coatings have been gradually phased out, stimulating the development of water-based intumescent coatings in recent years [27,28,29]. Moreover, waterborne intumescent coatings have significantly improved in adhesion and durability, gradually surpassing solvent-based coatings [30]. They also offer better safety during application and show greater stability, especially in high-humidity environments [31]. Waterborne intumescent fire-retardant coatings are considered to be the future development trend of coatings due to their environmentally friendly characteristics [32].

To enhance the fire protection of flammable substrates and ensure structural stability under complex conditions, there are increasing demands on the fire-retardant, thermal insulation, and mechanical properties of waterborne intumescent fire-retardant polymer composite coatings [33,34,35]. Unfortunately, as the fire-retardant performance increases, the thickness of the coating also significantly increases [36]. This is primarily due to the inadequate fire resistance, limited char strength, low expansion ratio, and insufficient water resistance of waterborne intumescent coatings [37]. Moreover, the majority of water-based polymer film-forming matrices are inherently flammable [38]. Therefore, functional fillers are often added to achieve rapid and effective fire extinguishing [39]. Generally, functional fillers are primarily composed of phosphorus-, nitrogen-, and silicon-containing compounds [40,41,42]. In addition, various inorganic nanoparticles, such as expanded graphite, carbon nanotubes (CNTs), graphene flakes, and montmorillonite (MMT), have been proven to impart superior fire retardancy to waterborne polymer materials [43,44,45]. Moreover, additives are essential for improving the quality and properties of waterborne intumescent fire-retardant polymer composite coatings. For instance, defoamers help to minimize bubble formation during the preparation process, and thickeners are used to modify the coating’s viscosity, enhancing its bonding and durability properties [46,47]. Thus, the development of novel waterborne film-forming matrix and the incorporation of various fillers, as well as additives, have led to the continuous emergence of diverse waterborne intumescent flame-retardant polymer composite coatings [48]. These components can produce synergistic effects in promoting carbonization, suppressing smoke, and enhancing weathering and corrosion resistance [49,50].

To date, several reviews have highlighted significant advancements in the preparation and fire-resistant effectiveness of intumescent coatings [51,52,53]. Unfortunately, comprehensive reviews on the design of waterborne intumescent fire-retardant polymer composite coatings and their impact on the overall performance of flammable materials are still lacking. This review will focus on the recent developments in the various components of waterborne intumescent fire-retardant polymer composite coatings, including intumescent flame-retardant systems, waterborne film-forming matrices, functional fillers, and additives, as well as discussing their effects on the overall performance of the coatings, such as flame retardancy, smoke suppression, thermal insulation, and mechanical properties. The typical intumescent flame-retardant mechanisms will also be discussed, followed by some practical industrial applications of waterborne intumescent flame-retardant polymer composite coatings. Finally, this review will address key challenges facing waterborne intumescent flame-retardant polymer composite coatings in the field of flame-retardant technology and propose future directions and opportunities for their development.

## 2. Formulation of Waterborne Intumescent Fire-Retardant Polymer Composite Coatings

Waterborne intumescent fire-retardant polymer composite coatings, which are used for protecting various types of flammable materials and are known as “firefighting suits”, should possess properties of viscosity, fluidity, and wettability to ensure that a uniform, stable, and solid fireproof protective layer can be formed, effectively playing a fireproof role when a fire occurs [54,55]. They are composed of chemicals that prevent materials from burning or slow down their rate of burning, primarily including intumescent fire-retardant systems (IFRs) (comprising an acid source, carbon source, and gas source), film-forming matrices, functional fillers, and additives [56]. The formulation of typical waterborne intumescent fire-retardant polymer composite coatings can be seen in Table 1. Notably, the waterborne coatings use water as the dispersion medium and water-based polymers as the film-forming material [57].

### 2.1. Intumescent Fire-Retardant Systems

**Figure 1:**
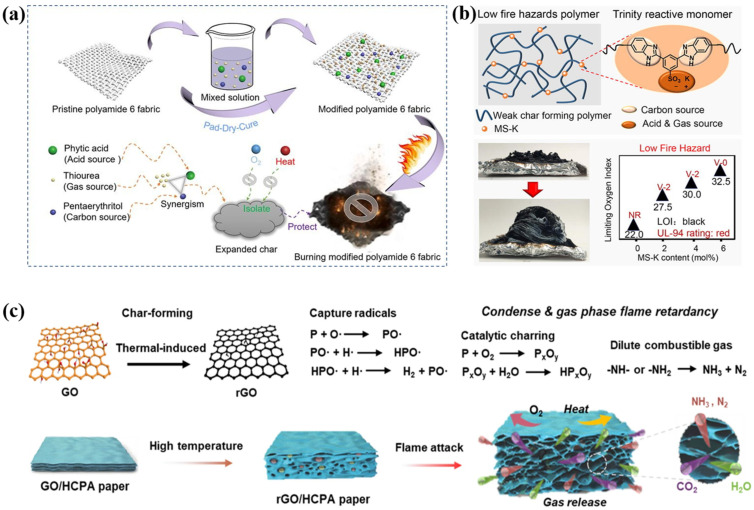
(**a**) The IFR systems consist of an acid source, a gas source, and a carbon source. Representative examples: (**a**) Phytic acid as the acid source, thiourea as the gas source, and pentaerythritol as the carbon source. (**b**) The sulfonate group works as both the acid and gas sources, while the benzimidazole group acts as the carbon source. (**c**) Graphene oxide, phosphorus atom, and nitrogen atom/amino functional groups act as the carbon source, acid source, and gas source in the GO/HCPA system, respectively. Adapted with permissions from Refs. [62,63,64]. Copyright 2022@Elsevier Publisher, Copyright 2022@Elsevier Publisher, Copyright 2022@Springer Nature, Copyright 2020@Royal Society of Chemistry, Copyright 2013@American Chemical Society, and Copyright 2021@Elsevier Publisher.

IFRs are composite flame retardants that are primarily composed of nitrogen and phosphorus [58,59]. In practical applications, to further enhance the flame retardancy of IFRs, appropriate amounts of silicon-based flame retardants, inorganic flame retardants, and nano flame retardants are also introduced. The IFR is the primary component responsible for fire protection, as it forms an expanded barrier layer at high temperatures, blocking the heat conduction of flames as well as oxygen to the flammable substrate [60,61]. The IFR is mainly composed of three parts: an acid source, a carbon source, and a gas source, which act as a dehydrating agent, charring agent, and foaming agent, respectively (Figure 1) [62,63,64].

Common acid sources include boric acid, phosphoric acid, and ammonium polyphosphate [65,66,67]. Upon heating, these compounds decompose to produce inorganic acids, which subsequently react with the carbon source through a dehydration esterification reaction, resulting in the formation of a molten carbon layer [68,69]. Common carbon sources, such as starch, chitosan, and polyols, are rich in carbon content, facilitating dehydration and charring processes that are essential for forming a porous intumescent carbon layer that effectively inhibits flame propagation and protects flammable substrates from further degradation and combustion [70,71,72]. Common gas sources include urea, melamine, and chlorinated paraffin [73,74,75]. Under high temperatures, gas sources undergo decomposition reactions, releasing large volumes of non-toxic, non-flammable gases such as ammonia, nitrogen, and carbon dioxide [76]. These gases cause the amorphous carbon layer to expand and foam, ultimately forming a porous intumescent carbon layer that serves as a thermal barrier [77]. In recent years, due to concerns about energy consumption and the promotion of green environmental protection concepts, IFRs based on biomaterials have received widespread attention. For example, biomaterials such as phytic acid, DNA, and proteins can serve as excellent carbon sources, acid sources, and gas sources [78,79,80].

To achieve excellent flame retardancy, the acid source, carbon source, and gas source should synergistically interact to form an expanded porous barrier layer with thermal and oxygen insulation functions [81]. The coordination of the decomposition temperatures of each component is a critical influencing factor. For instance, if there is a significant discrepancy between the decomposition temperatures of the carbon source and the acid source, or if the gas source decomposes in advance, the intumescent flame-retardant effect will not be achieved. Additionally, the IFR system faces challenges related to uniform dispersion, compatibility, and water resistance when utilizing water dispersion media and water-based film-forming materials.

### 2.2. Waterborne Film-Forming Matrices

Film-forming matrices are the main components of intumescent fire-retardant polymer composite coatings, enabling all components to adhere to the substrate surface to form a continuous film [82]. Due to their ability to avoid releasing large amounts of toxic and harmful gases during combustion, waterborne film-forming matrices are currently popular and are expected to replace solvent-based film-forming matrices [83].

The properties of waterborne film-forming matrices are crucial for achieving effective intumescence and flame retardancy in coatings. As is well known, to meet the requirements of normal working conditions, the waterborne film-forming matrix must possess excellent weather resistance and stability [84]. In addition, to ensure the adhesion strength and reliability of the coating on flammable substrates in complex environments, the waterborne film-forming matrices should exhibit excellent adhesion, fire resistance, and water resistance [85]. Some waterborne film-forming matrices can also serve as carbon sources, where their viscosity, melting temperature, and pyrolysis temperature need to be compatible with the pyrolysis reaction of the acid source to ensure the production of a uniform and compact intumescent carbon layer [86]. At present, acrylic resins, epoxy resins, polyurethanes, and silicone resins are commonly used waterborne film-forming matrices [87,88,89,90].

#### 2.2.1. Waterborne Acrylic-Resin-Based Intumescent Fire-Retardant Coatings

**Figure 2:**
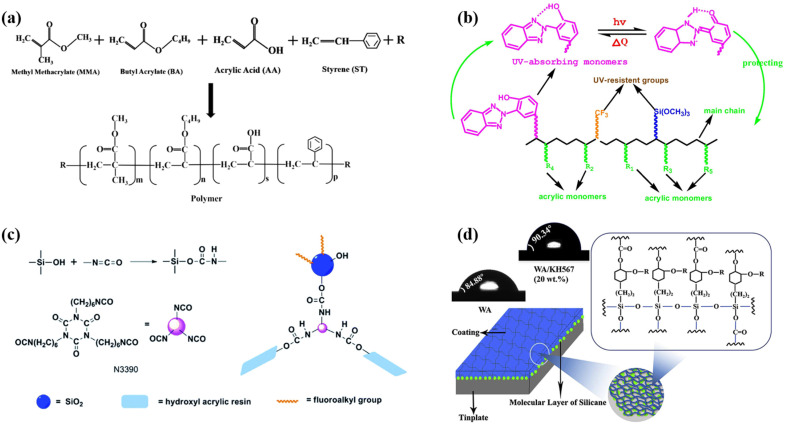
Composition and modification of waterborne acrylic resin: (**a**) Structure of waterborne acrylic resin and its monomers. (**b**) Fluorine–silicone modification. (**c**) Polyisocyanate crosslinking modification. (**d**) 2-(3,4-Epoxy) ethyltriethoxysilane modification. Adapted with permissions from Refs. [91,96,97,98]. Copyright 2021@American Chemical Society, Copyright 2018@Elsevier Publisher, Copyright 2015@Royal Society of Chemistry, Copyright 2020@Elsevier Publisher.

Acrylic resins are typically copolymerized using methyl methacrylate, acrylic acid, butyl acrylate, styrene, and acrylate monomers (Figure 2a) [91]. Waterborne acrylic resins, which disperse well in water, are obtained by introducing hydrophilic groups during the synthesis of acrylic resins. However, the challenges of poor water resistance, inadequate corrosion resistance, and lack of compactness limit their applications in intumescent fire-retardant coatings. Consequently, the modification of waterborne acrylic resins has garnered significant attention in recent decades.

The modification strategies for waterborne acrylic resins include fluorine-containing organic modification, polyurethane modification, silicone modification, and epoxy resin modification [92,93,94,95]. Lei et al. designed novel acrylic resin polymers by applying 2-[3-(2H-benzotriazol-2-yl)-4-hydroxyphenyl] ethyl methacrylate, vinyltrimethoxysilane, and hexafluorobutyl methacrylate. This fluorine–silicone acrylic resin can maintain inherent adhesion properties and high mechanical strength while enhancing the water and weather resistance of the coatings (Figure 2b) [96]. Xue et al. carried out research to investigate the effects of fluoroalkyl silane-modified silica nanoparticles and polyisocyanate on the hydrophobic properties and mechanical performance of acrylic polyurethane paints (Figure 2c) [97]. The results of the water contact angle measurements suggest that the acrylic polyurethane coatings exhibit excellent superhydrophobic abilities. This can be attributed to the micro- and nano-scale roughness structures constructed by hydrophobic silica nanoparticles. The hammer-impact or cross-cutting tests showed that the coating could remain intact, indicating superior mechanical strength. Jiao et al. utilized 2-(3,4-epoxy) ethyltriethoxysilane to modify waterborne acrylic resins. The results suggest that the resin subjected to the silicone treatment exhibited exceptional hydrophobicity and mechanical properties (Figure 2d) [98].

**Figure 3:**
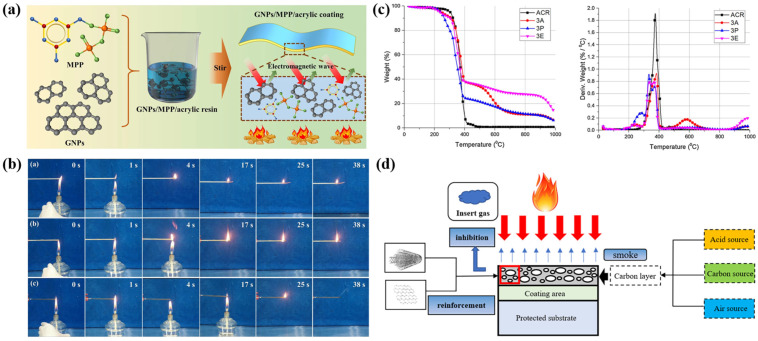
Enhancement of the flame retardancy of waterborne acrylic resin by combining other materials or adjusting formulations: (**a**) Preparation of waterborne acrylic-resin-based intumescent flame-retardant coatings using melamine polyphosphate (MPP) as an intumescent flame retardant, graphite nanoplates (GNPs) as a synergistic flame retardant/conductive filler, and acrylic resin as a film-forming agent, showing (**b**) excellent flame-retardant performance. (**c**) Improving flame-retardant effects by adjusting the ratio of traditional flame-retardant additives. (**d**) Mixed carbon materials were applied as fire-retardant filler in the waterborne acrylic-resin-based intumescent flame-retardant coatings. Adapted with permissions from Refs. [99,100,101]. Copyright 2021@Springer, Copyright 2021@Elsevier Publisher, Copyright 2021@Elsevier Publisher.

To enhance the flame-retardant properties of waterborne acrylic-resin-based intumescent fire-retardant coatings, integrating them with other materials is an effective approach. Liang et al. developed waterborne acrylic-resin-based intumescent fire-retardant coatings by using melamine polyphosphate and graphite nanoplates (Figure 3a) [99]. The aim of this study was to investigate the potential synergistic flame retardancy when combining melamine polyphosphate with graphite nanoplates. The results demonstrated that the incorporation of melamine polyphosphate and graphite nanoplates into acrylic resin could markedly improve the fire resistance of the coatings (Figure 3b). Ng et al. formulated eco-friendly fire-resistant acrylic coatings by varying the ratios of ammonium polyphosphate, pentaerythritol, and expandable graphite to modify their thermal and fire resistance performance. The results indicated that among ammonium polyphosphate, pentaerythritol, and expandable graphite, a higher loading of expandable graphite in the coatings demonstrated the best performance (Figure 3c) [100]. Zhan et al. conducted an investigation to analyze the impacts of mixed carbon materials, such as carbon nanotubes and graphene, on the fire-retardant properties of waterborne acrylic-resin-based intumescent fire-retardant coatings (Figure 3d) [101]. The findings revealed that the synergistic interaction significantly improved fire protection and thermal stability, which could be attributed to the combination of graphene and carbon nanotubes. The application of a 3:1 weight ratio of graphene to carbon nanotubes led to a significant reduction in the peak heat release rate (PHRR), total heat release (THR), and time to ignition (TTI), with values of 32.29 kW·m^−2^, 1.13 MJ·m^−2^, and 143 s, respectively. As a result, the waterborne acrylic-resin-based intumescent fire-retardant coatings protected the underlying substrate during combustion, thereby reducing the amount of smoke released.

#### 2.2.2. Waterborne Epoxy-Resin-Based Intumescent Fire-Retardant Coatings

Epoxy resins, a class of polymers containing multiple epoxy functional groups, were first synthesized in the early 20th century [102]. In particular, waterborne epoxy resins are widely used as film-forming matrices for intumescent fire-retardant coatings due to their excellent adhesion and chemical resistance [103]. Typically, waterborne epoxy resin coatings are composed of two main components: waterborne epoxy resin dispersions, and amine curing agents [104]. Currently, phase inversion and chemical modification are the main methods for preparing waterborne epoxy resin dispersions [105].

**Figure 4:**
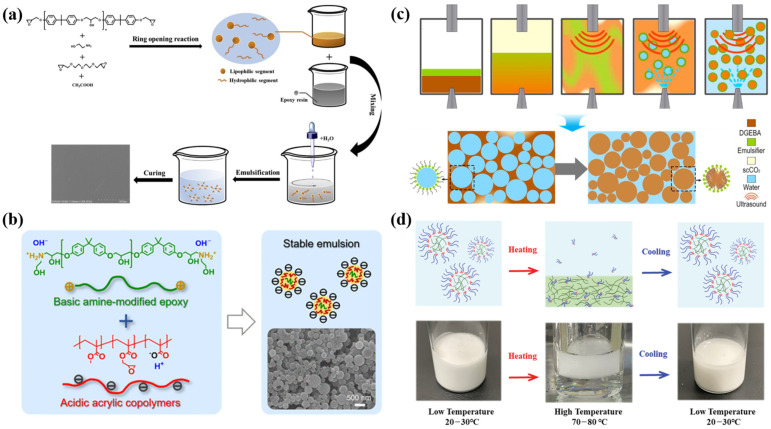
The principal methods for preparing waterborne epoxy dispersions include (**a**) phase inversion, (**b**) epoxy chemical modification, (**c**) ultrasound-assisted supercritical carbon dioxide (scCO_2_) technology, and (**d**) amphiphilic poly(hydroxyaminoethers) emulsifiers. Adapted with permissions from Refs. [106,107,108,109]. Copyright 2019@Elsevier Publisher, Copyright 2024@American Chemical Society, Copyright 2021@Elsevier Publisher, Copyright 2021@Wiley.

The phase inversion method involves converting a W/O (water-in-oil) system into an O/W (oil-in-water) system with the assistance of high-speed shear forces [106]. In addition, to overcome the poor stability of the dispersion caused by the wide particle size distribution, an emulsifier needs to be used in the waterborne epoxy resin dispersion. For example, Zhan et al. constructed a nanometer-scale and stable waterborne epoxy resin dispersion by employing an emulsifier composed of polyethylene glycol diglycidyl ether and glacial acetic acid (Figure 4a) [105]. Finally, the as-prepared waterborne epoxy resin dispersions were used to prepare waterborne epoxy resin coatings. The results indicate that these coatings exhibit excellent thermal stability and mechanical properties.

The chemical modification method involves the incorporation of hydrophilic groups into the epoxy resin structure, thereby enabling its emulsification in water. For example, Chen et al. introduced ethanolamine to modify the end groups of epoxy resin, providing alkaline conditions to promote the dissociation in water (Figure 4b) [107]. Due to electrostatic stabilization, successful emulsification can be achieved without additional dispersants. As a result, the waterborne epoxy dispersion, as a precursor to prepare coatings for the protection of steel surfaces, exhibits excellent corrosion resistance. In addition, Gao et al. studied the preparation of waterborne dispersions of epoxy resin using an ultrasonic-assisted supercritical CO_2_ nanoemulsification technique (Figure 4c) [108]. Huang et al. successfully stabilized epoxy resins in water using amphiphilic poly(hydroxyaminoethers) (Figure 4d) [109].

**Figure 5:**
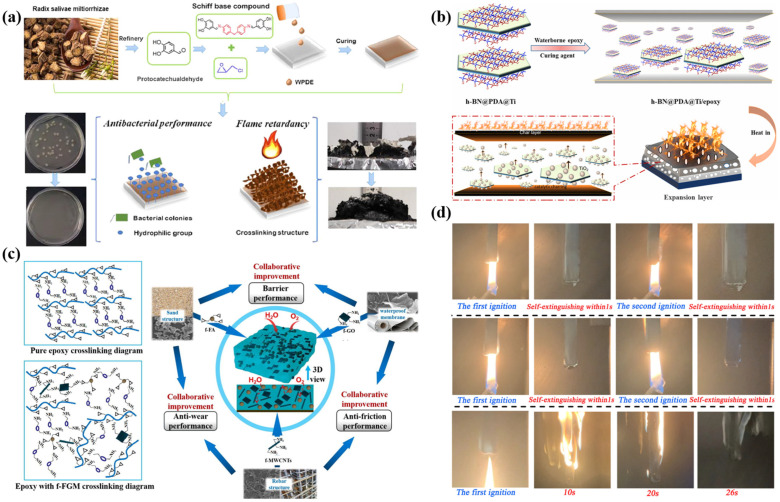
Methods to enhance the performance of waterborne epoxy resins: (**a**) structural design, (**b**) grafting, (**c**) blending, and (**d**) optimization of curing agent content. Adapted with permissions from Refs. [111,112,113,114]. Copyright 2022@Elsevier Publisher, Copyright 2021@Elsevier Publisher, Copyright 2021@Elsevier Publisher, Copyright 2024@MDPI.

To optimize the comprehensive performance (especially the flame retardancy) of waterborne epoxy-resin-based intumescent fire-retardant coatings, special structural design, graft modification, blending modification, and curing agent content regulation are effective approaches [110]. Ji et al. developed a facile strategy to introduce a Schiff base structure into waterborne epoxy resin (Figure 5a) [111]. After the curing reaction, the treated waterborne epoxy thermoset coatings exhibited superior flame retardancy. Compared to the unmodified epoxy resin coatings, the PHRR and THR values of Schiff base waterborne epoxy resin coatings decreased by 57.3% and 25.6%, respectively. Yang et al. developed a waterborne epoxy resin intumescent fire-retardant coating for steel plates using modified hexagonal boron nitride as a flame-retardant component (Figure 5b) [112]. The coated steel plates exhibited improved strength and oxidation resistance properties. Wang et al. fabricated waterborne epoxy resin protective coatings composed of stacking fly ash, graphene oxide, and multi-walled carbon nanotubes grafted with a silane coupling agent to enhance the anti-corrosion performance and wear resistance (Figure 5c) [113]. Yang et al. studied the effects of different diluents and curing agents on the flame retardancy of epoxy-resin-based coatings. The results showed that 1,4-butanediol diglycidyl ether and T403 improved the fire retardancy and smoke suppression properties of the coating. Specifically, the peak heat release rate (PHRR), total heat release (THR), total smoke production (TSP), and peak smoke production rate (PSPR) were reduced by 13.15%, 13.9%, 17.45%, and 5.48%, respectively (Figure 5d) [114].

#### 2.2.3. Waterborne Polyurethane-Based Intumescent Fire-Retardant Coatings

**Figure 6:**
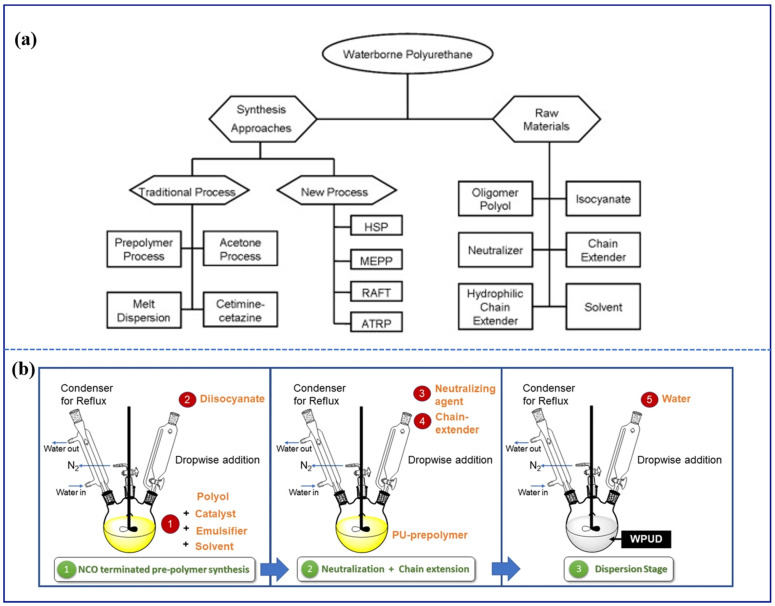
(**a**) The synthesis method of waterborne polyurethane, the required raw materials, and (**b**) the synthesis route. Adapted with permissions from Refs. [116,117]. Copyright 2015@Elsevier Publisher, Copyright 2022@Springer.

Waterborne polyurethane coatings possess the advantage of curing at room temperature, providing superior flexibility compared to epoxy resin coatings, as well as enhanced aesthetics and weather resistance [115]. The raw materials and methods for synthesizing waterborne polyurethane are summarized in Figure 6a [116]. Specifically, polyol, isocyanate, chain extenders (difunctional low-molecular chain extenders and hydrophilic chain extenders), water, and neutralizers are the raw materials used in synthesizing waterborne polyurethane. It is worth noting that the structure of waterborne polyurethane is divided into hard segments and soft segments, which are composed of isocyanate and polyol, respectively. Traditional processes for obtaining waterborne polyurethane include the acetone process, prepolymer emulsification, cetimine–cetazine processes, and melt dispersion. Among these, the acetone process and prepolymer emulsification are the most widely used. In addition to these conventional methods, researchers have recently developed other techniques to synthesize novel waterborne polyurethane, such as homogeneous solution polymerization (HSP), the miniemulsion polymerization process (MEPP), addition–fragmentation chain transfer (RAFT) polymerization, and atom transfer radical polymerization (ATRP). In summary, the synthesis of waterborne polyurethane involves three main stages: the first stage is the synthesis of NCO-terminated pre-polymer, the second stage involves neutralization and chain extension, and the final stage is the dispersion of the synthesized pre-polymer in water through phase inversion (Figure 6b) [117].

**Figure 7:**
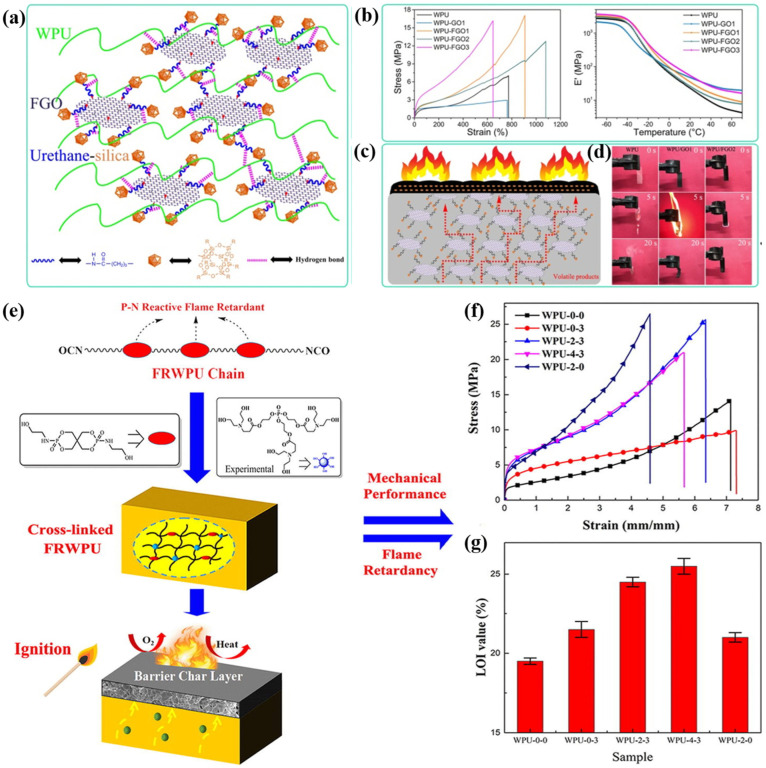
The approaches to modifying waterborne polyurethane: (**a**–**d**) incorporating fillers into the polyurethane matrices, and (**e**–**g**) modifying the basic building blocks of polyurethane with functional monomers. Adapted with permissions from Refs. [118,119]. Copyright 2019@Elsevier Publisher, Copyright 2019@Elsevier Publisher.

The methods for enhancing the flame retardancy of waterborne polyurethane coatings can be classified into additive types and reactive types. The additive method, which involves directly incorporating flame-retardant components into the waterborne polyurethane matrix, is widely employed due to its simplicity and operational flexibility. For example, Du et al. used this strategy to construct multifunctional waterborne polyurethane coatings, where urethane–silica-functionalized graphene oxide (FGO) was alternatively incorporated on a waterborne polyurethane matrix (Figure 7a) [118]. With 2 wt.% FGO, the tensile strength and elongation-at-break of the coatings were 17.1 MPa and 925.0%, respectively. Additionally, the flammability of the coatings was significantly reduced, with an UL-94 rating of V-2. The reactive approach involves the synthesis of waterborne polyurethane by grafting or incorporating flame-retardant elements or groups into the polyurethane molecular chain, which maintains the inherent flexibility of the polyurethane coating. Wang et al. synthesized flame-retardant waterborne polyurethane by conjugating tri (N,N-bis-(2-hydroxyethyl) acyloxoethyl) phosphate (TNAP) with pentaerythritol di-N-hydroxyethyl phosphamide (PDNP) (Figure 7b) [119]. The prepared waterborne polyurethane exhibited superior flame retardancy and mechanical performances, with an LOI value of 25.5% and a tensile strength of 15.81 MPa.

#### 2.2.4. Waterborne Silicone-Based Intumescent Fire-Retardant Coatings

Silicone polymers or silicone-modified polymers serve as film-forming matrices in silicone-based intumescent fire-retardant coatings, offering superior flame retardancy, heat resistance, and hydrophobicity compared to other polymer coatings [120,121]. Recently, waterborne silicone coatings, especially those based on silicone emulsions, have been extensively studied and applied due to their remarkable environmental friendliness [122]. Based on the emulsifiers used during preparation, silicone emulsions can be classified into anionic, cationic, non-ionic, and complex ionic types [123].

**Figure 8:**
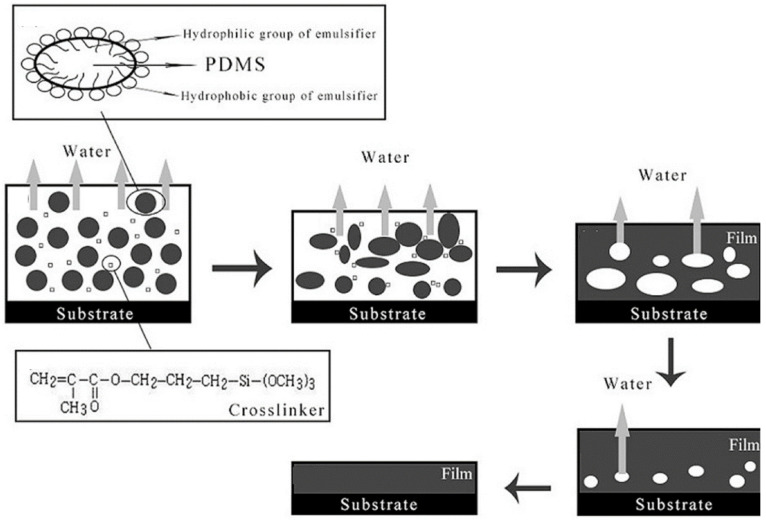
Schematic illustration of silicone emulsion film formation process. Adapted with permissions from Ref. [124]. Copyright 2020@MDPI.

The film formation mechanism of waterborne silicone coatings comprises both physical and chemical processes [124]. Initially, in waterborne silicone coating systems, as the water evaporates, the droplets move closer to each other and deform, resulting in physical film formation. Subsequently, a curing agent reacts with the silicone matrix, initiating a crosslinking reaction that forms a three-dimensional network structure, resulting in chemical film formation. For instance, Liu et al. summarized the film formation mechanism of a three-component waterborne silicone emulsion, as shown in Figure 8 [124]. The chemical film formation of the silicone emulsion occurred during water evaporation due to the slow rate of water evaporation. When the curing agent contacted the silicone emulsion particles, a crosslinking reaction took place within the silicone matrix. Under the influence of capillary pressure, the silicone emulsion particles gradually moved closer together as the water evaporated, and they eventually formed a uniform film.

**Figure 9:**
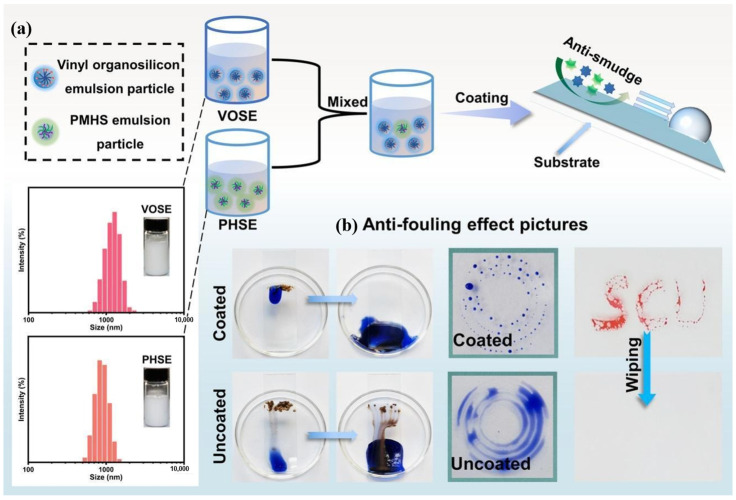
(**a**) Procedure for preparing waterborne silicone coating; (**b**) self-cleaning and anti-fouling properties of the coating. Adapted with permissions from Ref. [125]. Copyright 2023@Elsevier Publisher.

Designing an appropriate emulsification system is a crucial strategy for preparing high-performance waterborne silicone coatings. Li et al. utilized two reactive organosilicon surfactants in combination with corresponding anionic surfactants to emulsify polymethylhydrosiloxane and vinyl polysiloxane, subsequently curing them through a hydrosilylation reaction to obtain waterborne organosilicon coatings for glass (Figure 9b) [125]. The results indicated that the glass coated with the shielding layer exhibited excellent self-cleaning and anti-fouling performance (Figure 9b) [125]. Dong et al. employed silicone emulsions to successfully prepare thermally stable, antioxidant, and flame-retardant coatings, and the carbon residue increased significantly at high temperatures [126].

In summary, waterborne acrylic and waterborne epoxy resins are widely used as film-forming matrices during the formulation of intumescent fire-retardant polymer composite coatings due to their low cost and mature synthesis technology. However, waterborne acrylic resins have disadvantages such as long drying times, unstable performance at high and low temperatures, and inherent flammability. Waterborne epoxy resin coatings also have shortcomings in terms of weather resistance and corrosion resistance. Waterborne polyurethane and silicone offer better resistance to high and low temperatures, wear resistance, and good bonding properties with other materials, making them suitable for the protection of special flammable materials. Additionally, they can be used as hybrid emulsions to improve the properties of the coatings.

### 2.3. Functional Fillers

Functional fillers have been extensively studied as an effective element to improve the comprehensive performance of waterborne intumescent fire-retardant polymer composite coatings [127]. Generally, functional fillers serve as either pigments or carbon-forming agents to endow the coatings with enhanced water resistance, weather resistance, flame retardancy, and smoke suppression [57]. Until now, the functional fillers frequently employed in waterborne intumescent fire-retardant polymer composite coatings have been mainly divided into two types: inorganic fillers and organic fillers, which are discussed in detail below.

#### 2.3.1. Inorganic Fillers

Inorganic fillers, such as metal oxides, metal hydroxides, silicon compounds, montmorillonite, and carbon-based materials, are becoming increasingly popular as widely used functional fillers due to their low cost, eco-friendliness, and effectiveness [128]. For example, they have been used to treat polymer coatings to reduce thermal conductivity and improve flame retardancy by decreasing heat conductivity.

**Figure 10:**
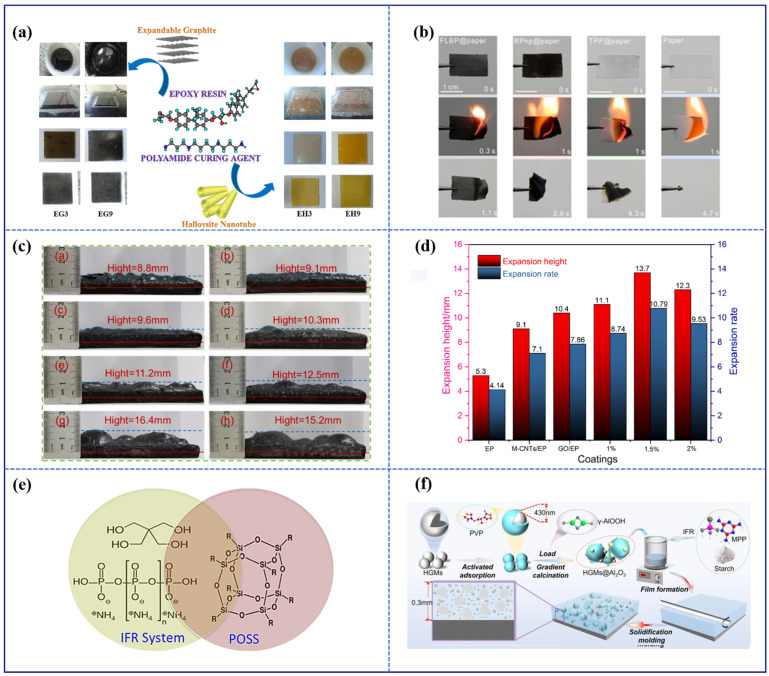
Common fillers for enhancing coating performance: (**a**) inorganic materials, (**b**–**d**) two-dimensional materials, (**e**) organic materials, and (**f**) organic/inorganic composites. Adapted with permissions from Refs. [112,129,130,131,132,133]. Copyright 2018@Elsevier Publisher, Copyright 2023@MDPI, Copyright 2021@Elsevier Publisher, Copyright 2022@Elsevier Publisher, Copyright 2018@Elsevier Publisher, Copyright 2024@Elsevier Publisher.

Recently, Vahabi et al. synthesized a functional filler composed of halloysite nanotubes and expandable graphite, which showed an excellent synergistic effect in improving the thermal degradation of epoxy resin coatings (Figure 10a) [129]. A low concentration of 3 wt.% halloysite nanotubes and 9 wt.% expandable graphite effectively prevented the coating’s flammability. Zhang and coworkers conducted a study to examine the impact of few-layer black phosphorus, red phosphorus nanoparticles, and triphenyl phosphate as flame-retardant fillers on the fire resistance performances of coatings [130]. As shown in Figure 10b, combustion in paper coated with few-layer black phosphorus, red phosphorus, and triphenyl phosphate extinguished after approximately 1.1 s, 2.9 s, and 4.3 s, respectively, whereas the uncoated paper burned continuously until it was completely consumed. Yang and coworkers developed a novel flame-retardant formulation with excellent oxidation resistance capabilities (Figure 10c) [112]. They utilized the synergy between organic titanium and polydopamine on boron nitride, demonstrating that the synergistic sample significantly enhanced the strength and oxidation resistance of the char layer, thereby improving the barrier effect to heat and oxygen. The expansion ratio and residual char increased compared to the intumescent formulation without fillers. Chen and coworkers constructed an intumescent coating composed of carbon nanotubes and graphene oxide. The hybrid coatings incorporating both carbon nanotubes and graphene oxide demonstrated superior fire-retardant performance compared to those containing only carbon nanotubes or graphene oxide (Figure 10d) [131].

#### 2.3.2. Organic Fillers

Organic fillers are primarily utilized in waterborne intumescent fire-retardant polymer composite coatings to enhance the construction and expansion of carbon layers. Therefore, organic fillers mainly consist of high-carbon-content substances. Additionally, organosilicon compounds, containing both silicon and carbon elements, have garnered widespread attention as organic fillers due to their unique structure, heat resistance, and flame retardancy. For example, dispersing polyhedral oligomeric silsesquioxane (POSS) nanoparticles into polymer coatings can increase their strength and thermal resistance while reducing their flammability (Figure 10e) [132].

In addition, to further improve the flame-retardant efficiency of waterborne intumescent fire-retardant polymer composite coatings, inorganic fillers are usually combined with organic fillers to enhance the synergistic effect. For example, Wang et al. constructed an intumescent coating using boehmite sol, hollow glass microspheres (HGMs), and polyvinylpyrrolidone (PVP) (Figure 10f) [133]. The composite coatings remarkably decreased the PHRR and effectively preserved the integrity of the intumescent layer during the combustion of the substrate materials. This superior performance was attributed to the thermal shielding effect of Al_2_O_3_ and HGMs, as well as the char layer formed from the degradation of PVP.

Furthermore, with the advancement of nanotechnology, various novel nanoparticles have been synthesized and are being utilized as functional fillers to enhance the mechanical and fire-resistant properties of intumescent coatings. For example, recent research has focused on the development of innovative two-dimensional nanofillers, including graphene, MXene, and metal–organic frameworks (MOFs), which are being increasingly incorporated into intumescent coatings due to their superior physicochemical properties [134,135,136].

### 2.4. Additives

Additives play a vital role in regulating the performance and condition of waterborne intumescent fire-retardant polymer composite coatings. They serve various functions and can be categorized primarily as defoamers and thickeners. Thickeners adjust the coating’s viscosity to enhance its stability and adhesion, whereas defoamers help minimize bubble formation during the coating preparation process.

**Figure 11:**
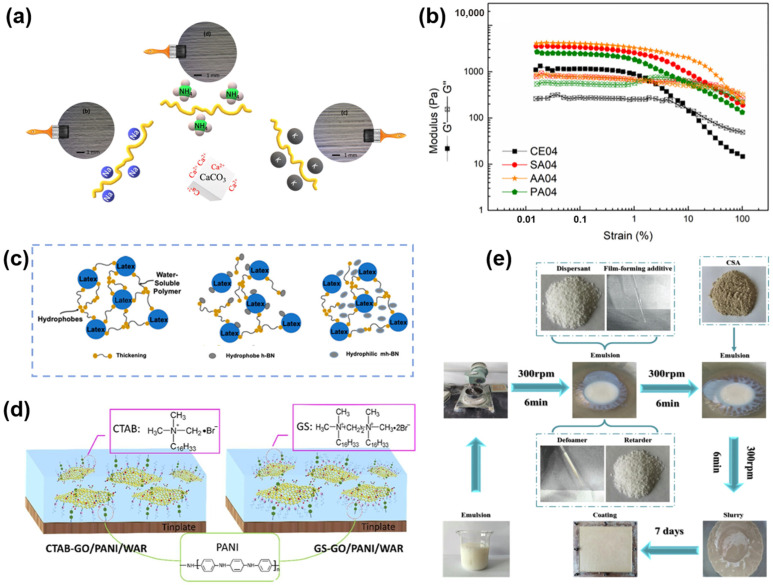
The role of additives in intumescent flame-retardant coatings: (**a**) Sodium alginate as a thickener, and (**b**) its viscoelasticity. (**c**) Hexagonal boron nitride nanosheets to improve coating stability. (**d**) Surfactant-modified coatings. (**e**) The synergistic effects of defoamers and film-forming additives on coating performance. Adapted with permissions from Refs. [137,138,139,140]. Copyright 2022@Elsevier Publisher, Copyright 2020@MDPI, Copyright 2021@Elsevier Publisher, Copyright 2021@Elsevier Publisher.

For example, Gaggero et al. proved that alginate is suitable as a thickener for waterborne coatings (Figure 11a) [137]. According to the characterization results of the coating formulation containing alginate as a thickener, the alginate samples exhibited higher shear-thinning behavior, which is beneficial for the stability and applicability of the coating formulation (Figure 11b) [137]. Ysiwata-Rivera et al. added hexagonal boron nitride (h-BN) nanosheets as a thickener to improve the stability and viscosity of the coating (Figure 11c) [138]. This improvement was attributed to the better compatibility of h-BN particles with the polymer coating matrix. Moreover, Wang et al. have reported that the properties of polymer coatings incorporated with cationic Gemini surfactants/graphene oxide/polyaniline outperform those of pure polymer coatings (Figure 11d) [139]. Liang et al. observed that the addition of appropriate defoaming agents could effectively remove internal defects in the coating, resulting in higher mechanical properties and improved water resistance (Figure 11e) [140].

## 3. Intumescent Flame-Retardant Mechanism

**Figure 12:**
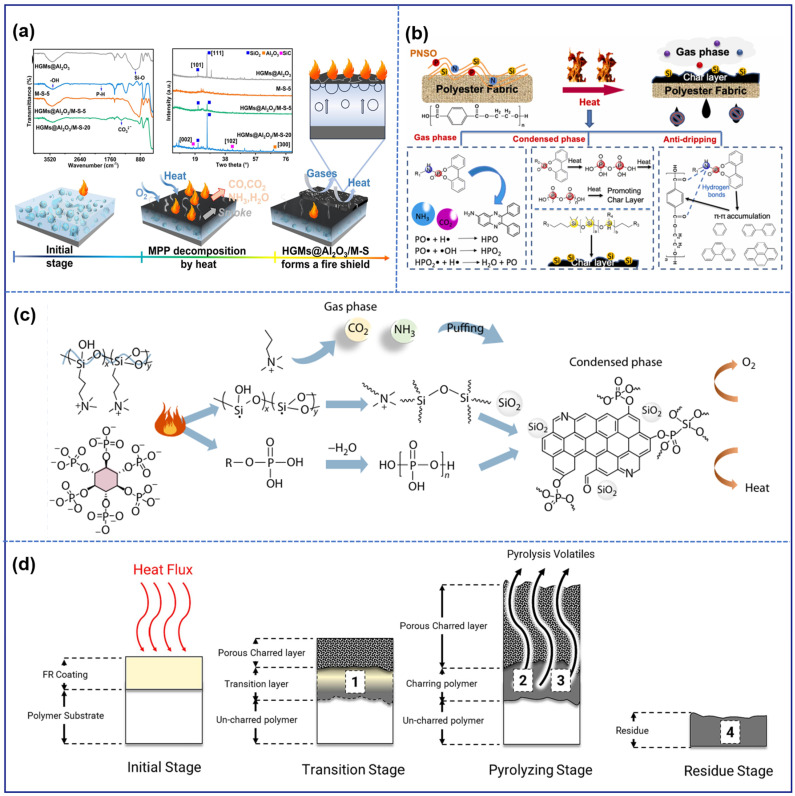
Intumescent flame-retardant mechanism: (**a**) The synergistic flame-retardant mechanism of hollow glass microspheres (HGMs) combined with melamine polyphosphate and starch (M-S system). (**b**) Phosphorus–nitrogen-containing silicone oil (PNSO) intumescent coating on polyester fabric. (**c**) Phytic acid sodium salt hydrate combined with N-[3(trimethoxysilyl)propyl]-N,N,N-trimethylammonium chloride as a coating. (**d**) Flame-retardant chemistry in the pyrolysis zone. Adapted with permissions from Refs. [133,142,143,144]. Copyright 2024@Elsevier Publisher, Copyright 2024@Elsevier Publisher, Copyright 2024@Springer, Copyright 2024@Elsevier Publisher.

Combustion is an extremely complex thermal oxidation process, typically accompanied by exothermic reactions, photon emission, and the formation of gas-phase and condensed-phase products [141]. The occurrence of combustion necessitates the presence of three essential components: a combustible substrate, an oxidizing agent (such as atmospheric air, molecular oxygen, or other oxidizers), and an ignition source (such as a flame or elevated temperature). These components must coexist and interact directly to initiate and sustain combustion. The absence of any of these components, or a failure in their interaction, will prevent the onset of combustion or lead to its immediate cessation. The intumescence process in coatings under thermal exposure can be classified into two primary mechanisms: the chemical mechanism, and the physical mechanism. The chemical mechanism encompasses a series of reactions, including the decomposition of an acid source, char formation, and the release of non-combustible gases. In contrast, the physical mechanism involves structural changes in the material, such as melting, foaming, and expansion. The prevailing understanding of the fire-retardant mechanisms of waterborne intumescent fire-retardant polymer composite coatings is that fire resistance is achieved through interactions in both the condensed phase and the gas phase. Additionally, the char layer serves to slow down the transfer of heat and oxygen between these phases. Figure 12 illustrates the fire-retardant mechanisms of several typical intumescent fire-retardant polymer composite coatings [133,142,143,144].

### 3.1. Condensed-Phase Mechanism

Delaying or preventing thermal decomposition of materials in the solid phase, which contributes to fire resistance, is classified as condensed-phase flame retardancy [145]. When the materials are attacked by flames, the intumescent fire-retardant systems undergo three reaction stages: esterification, dehydration, and carbonization, ultimately forming porous and dense char layers [146]. The generation of inorganic acid by an acid source is the initial step, followed by the catalysis of a carbon source, resulting in the construction of the char layer. As a result of the presence of abundant functional groups in the chemicals, the carbon source undergoes rapid decomposition and carbonizes at elevated temperatures, forming a char layer with a carbon-rich skeleton. Finally, under combustion conditions, inert and non-flammable gases are released by the gas source, leading to the expansion of the char layer. The porous structure of the char layer is the main reason for the reduction in thermal conductivity. On the one hand, the char layer is capable of efficiently isolating the ignition source and oxygen; on the other hand, it extends the heat and mass transfer path between the gas phase and the condensed phase. Ultimately, the fire-retardant performance of the material is greatly enhanced, achieving the goal of safety and protection.

### 3.2. Gas-Phase Mechanism

The gas-phase flame-retardant mechanism involves inhibiting free radicals that contribute to chain reactions in the combustion process [147]. By interrupting free radical reactions, the heat release process can be slowed, and the pyrolysis of materials can also be partially hindered, ultimately resulting in the effective suppression of combustion. During the combustion process, the flame retardants in the intumescent system undergo thermal decomposition to produce inert gases such as CO_2_, NH_3_, and N_2_ [148]. Consequently, the oxygen content and the concentration of flammable gases in the combustion atmosphere are significantly reduced. In addition, the flame retardant absorbs heat during the chemical decomposition process, reducing the reaction temperature and alleviating the spread of combustion, thereby enhancing the flame-retardant effect.

## 4. Applications of Waterborne Intumescent Fire-Retardant Polymer Composite Coatings

**Figure 13:**
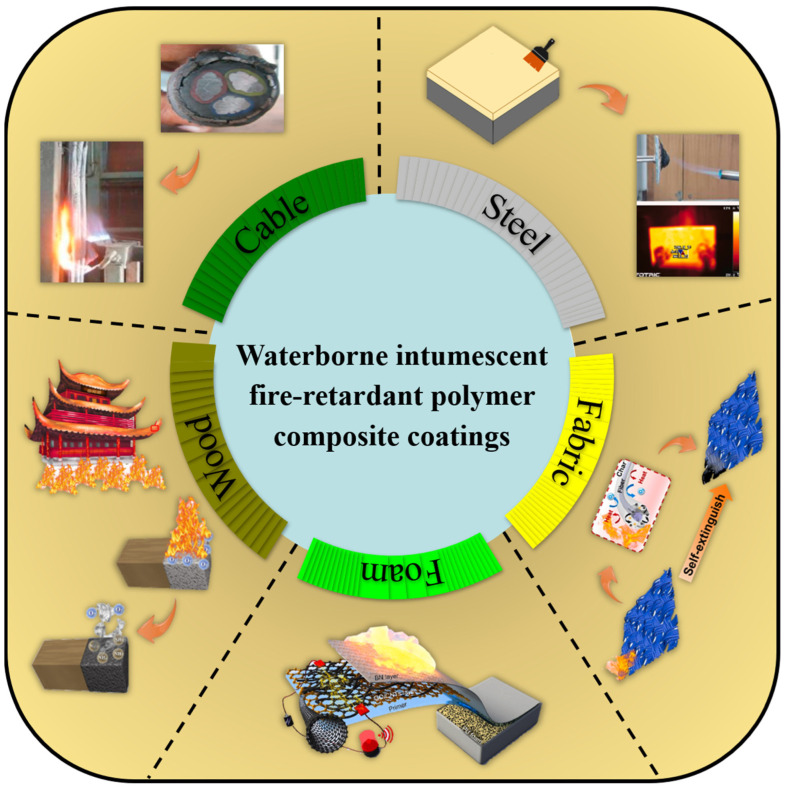
Industrial applications of intumescent flame-retardant coatings. Adapted with permissions from Refs. [99,149,150,151,152]. Copyright 2020@Elsevier Publisher, Copyright 2023@American Chemical Society, Copyright 2022@Elsevier Publisher, Copyright 2021@Springer, Copyright 2021@Elsevier Publisher.

In this section, we explain and discuss the protective applications of coatings based on waterborne intumescent fire-retardant polymer composites on various materials, including wood, foam, fabric, steel, cable, and so forth. Each of these functions can be attributed to the exceptional characteristics of the waterborne intumescent fire-retardant polymer composite coating itself, such as being green, environmentally friendly, highly fireproof, stable, and weather-resistant, as well as other characteristics mentioned in the Introduction section (Figure 13).

### 4.1. Wood

Wood possesses abundant resources, sustainability, and robust mechanical properties, making it a widely utilized material in construction, interior decoration, furniture, and other fields since ancient times. However, the inherent flammability of wood presents significant fire hazards, thereby limiting its applications. Furthermore, considering that many traditional wooden structures are treasures of ancient civilizations, it is urgent to improve the flame-retardant properties of wood. Compared to traditional heat treatment methods, waterborne intumescent flame-retardant polymer composite coatings offer a more cost-effective and convenient approach to mitigating fire hazards while fully preserving the natural properties of wood materials. For instance, Liang et al. prepared a waterborne intumescent flame-retardant polymer composite coating composed of melamine polyphosphate (MPP) as the intumescent flame retardant, graphite nanoplates (GNPs) as the synergistic flame retardant/functional filler, and acrylic resin as the film-forming matrix [99]. The coatings demonstrated excellent flame retardancy and effective heat resistance, and they can be used for wood protection.

### 4.2. Foam

Foams have been extensively used in various areas, including packaging, automotive interiors, and building exteriors, due to their porous structure and superior properties such as low density, flexibility, and thermal insulation. However, once exposed to high temperatures or flames, these materials are prone to structural damage and easily ignite, causing the combustion to spread rapidly. More seriously, large amounts of smoke and toxic gases are released during the combustion process, severely endangering the safety of life and property. Modifying the surface of foams with waterborne intumescent flame-retardant polymer composite coatings is an effective way to isolate them from the continuous damage caused by fire and oxygen. More importantly, foams can still maintain their inherent mechanical properties. For example, Ma et al. reported that a waterborne intumescent flame-retardant polymer composite coating applied to the foam surface effectively achieved self-extinguishing and flame retardancy of the foam [149].

### 4.3. Fabric

Fabrics are widely used textile materials for protection against cold and maintaining warmth in daily life, due to their advantages of moisture absorption and breathability, low cost, wear resistance, high temperature resistance, and good biocompatibility. However, fabrics are rich in carbon, hydrogen, and oxygen elements, as a result of which they are flammable and can easily cause fire accidents. The application of waterborne intumescent flame-retardant polymer composite coatings represents one of the most effective and economical strategies for imparting flame retardancy to fabrics, making it highly suitable for industrial-scale implementation. For example, to manufacture fire-safe fabrics in an efficient and environmentally friendly way, Wang et al. designed a novel waterborne intumescent flame-retardant polymer composite coating and deposited it on the surface of fabrics. When exposed to flame, the fabrics exhibited excellent flame retardancy and self-extinguishing properties [150].

### 4.4. Steel

Steel structural materials are among the mainstream building components in both fundamental research and practical uses, due to their high flexibility for processing and integration, as well as their good mechanical properties. Although steel structures are inherently non-combustible, their high thermal conductivity, with a coefficient exceeding 50 W/(m·K), limits their ability to withstand elevated temperatures. Consequently, when exposed to flames, unprotected steel structures can heat up rapidly in a short period of time. Generally, they begin to suffer damage at temperatures between 450 and 500 °C, losing their basic load-bearing capacity and potentially leading to the collapse of buildings. For instance, Grenfell Tower in the UK suffered a devastating collapse in 2017 due to a fire, resulting in significant loss of life and property. Thus, applying waterborne intumescent fire-retardant polymer composite coatings to enhance the fire resistance of steel structures is crucial for building safety. For example, Zhou et al. employed a waterborne acrylic-resin-based intumescent fire-retardant coating containing organically modified glass fiber filler, ammonium polyphosphate (APP), melamine (MEL), and pentaerythritol (PER) on a steel structure [151]. The results indicated that the flame retardancy of the coated steel plate was twice that of the uncoated steel plate, demonstrating the effective protective capability of the waterborne intumescent fire-retardant coating for steel.

### 4.5. Cable

As carriers of electric energy transmission, cables are extremely important in industrial production, information transmission, and energy extraction. Essentially, the structure of the cable consists of three parts: the conductor core, the insulation layer, and the sheath layer. However, the insulation layer and the sheath layer of the cable are commonly constructed from flammable materials like rubber or plastic, which present significant hidden dangers and production risks. Most fire incidents in the past have involved cables. Therefore, it is crucial to study the flame-retardant modification of cables to improve their flame-retardant properties and reduce safety hazards such as fire. Considering green environmental protection and current flame-retardant technology, the main method for the flame-retardant modification of cables is to use waterborne intumescent flame-retardant polymer composite coatings. For example, Aurtherson et al. investigated the failure process of cables by using flames to attack cables coated with fire-retardant coatings. The results indicated that a specific coating thickness could significantly enhance the flame-retardant properties of the cables [152].

## 5. Conclusions and Perspective

The introduction of intumescent fire-retardant coatings on combustible materials is a promising strategy for enhancing their flame-retardant properties because of the efficient isolation of heat and oxygen during the combustion process. However, traditional intumescent fire-retardant coatings have the drawback of requiring large quantities of harmful organic solvents during their production, leading to significant environmental pollution and posing risks to human health. Furthermore, the impact of complex environmental conditions on material properties necessitates advancing intumescent fire-retardant polymer composite coatings with tailored properties to meet specific application requirements. Therefore, waterborne intumescent fire-retardant polymer composite coatings are extensively used in the protection of combustible materials, owing to their high performance, low toxicity, and minimal impacts on human health and the environment.

This review summarizes the state-of-the-art understanding of the composition and intumescent flame-retardant mechanisms for waterborne intumescent fire-retardant polymer composite coatings, focusing on the effects of intumescent flame-retardant systems, film-forming matrices, functional fillers, and additives on the flame retardancy, heat insulation, and mechanical properties of the resultant coatings. The available literature showcases both condensed-phase mechanisms and gas-phase mechanisms as potential mechanisms for waterborne intumescent fire-retardant polymer composite coatings. Additionally, we have provided an in-depth introduction of the application of these coatings on a variety of typical flammable and protected substrates. Continued research and development on waterborne intumescent fire-retardant polymer composite coatings and their applications in combustible materials present an opportunity for significant advancements in the performance and safety aspects of flame-retardant systems.

Although significant advancements have been made in advancing waterborne intumescent fire-retardant polymer composite coatings in recent years, substantial progress is still required in their rational design to satisfy the growing performance and safety requirements of flame-retardant systems. Future research should focus on several challenging yet significant areas that offer promising avenues for further exploration:

(1) Sustainability issues: Most flame retardants employed in the reported waterborne intumescent fire-retardant polymer composite coatings are derived from petroleum-based resources, posing significant long-term sustainability concerns. Additionally, their synthesis often necessitates extensive use of organic solvents, resulting in elevated production costs and potential environmental impacts, thereby constraining their practical industrial applications. Furthermore, a thorough evaluation of the environmental and human toxicity of these flame retardants is imperative.

(2) Optimization of coating thickness: Although many waterborne intumescent flame-retardant polymer composite coatings have demonstrated high flame-retardant efficiency, sufficient thickness is required. The effective thickness of these coatings generally depends on the ratio and synergy between the various components in the formulation. However, the current research predominantly focuses on fixed component ratios with limited interactions between them, significantly constraining their flame-retardant efficiency. Moreover, the interfacial interactions between the coating and the substrate, which play a crucial role in determining flame-retardant performance, remain insufficiently studied.

(3) Transparency of the coatings: Traditional waterborne intumescent fire-retardant polymer composite coatings are typically opaque, rendering them unsuitable for applications requiring visible aesthetics. Therefore, developing waterborne intumescent fire-retardant polymer composite coatings with adjustable transparency is essential for expanding their application range in the future.

In conclusion, further research and development are urgently needed in the rational design of waterborne intumescent fire-retardant polymer composite coatings for reliable flame-retardant technologies. The sustainability issues, optimization of coating thickness, and transparency of the coatings will be key areas to explore. These advancements will play a pivotal role in developing highly efficient flame-retardant strategies, effectively addressing the changing requirements for combustible materials across diverse applications.

## Figures and Tables

**Table 1:** Formulation of typical intumescent fire-retardant polymer composite coatings.

Components	Commonly Used Substances	Role
Acid source	H_3_BO_3_, H_3_PO_4_, [(NH4)_3_PO_4_]n	Dehydrating agent
Carbon source	starch, chitosan, polyol	Charring agent
Gas source	Urea, melamine, chlorinated paraffin	Foaming agent
Film-forming matrices	Acrylic resin, epoxy resin, polyurethane, silicone	Continuous phase produces coating
Functional fillers	Alumina, kaolin, diatomite, silica fiber	Improving flame resistance
Additives	Defoamer, thickener	Enhancing the condition and effectiveness of coatings
